# Optogenetically enhanced physical reservoir computing with *in vitro* neural networks for obstacle avoidance

**DOI:** 10.1117/1.JBO.30.10.105004

**Published:** 2025-10-22

**Authors:** Yin Deng, Jie Li, Yarong Lin, Zeying Lu, Lili Gui, Longze Sha, Xiaojuan Sun, Yueheng Lan, Qi Xu, Kun Xu

**Affiliations:** a Beijing University of Posts and Telecommunications, State Key Laboratory of Information Photonics and Optical Communications, Beijing, China; b Beijing University of Posts and Telecommunications, School of Electronic Engineering, Beijing, China; c Chinese Academy of Medical Sciences and Peking Union Medical College, State Key Laboratory of Common Mechanism Research for Major Diseases, Department of Biochemistry and Molecular Biology, Institute of Basic Medical Sciences, Beijing, China; d Beijing University of Posts and Telecommunications, School of Physical Science and Technology, Beijing, China

**Keywords:** optogenetic stimulation, neural networks, reservoir computing

## Abstract

**Significance:**

The effects of optogenetic stimulation (OS) on *in vitro* neural network behavior were studied through a reservoir computing-based obstacle avoidance task, revealing its impact on the task-processing capabilities of the network. Furthermore, it is demonstrated that a minimal output of signals from 15 neurons in the network is sufficient to achieve stable task control, with a success rate exceeding 95%. The optogenetically enhanced biological reservoir computing frame could find applications in neuro-robotic control and brain-inspired intelligence.

**Aim:**

We aim to utilize optogenetically controlled *in vitro* neural networks and the first-order reduced and controlled error (FORCE) learning algorithm to achieve obstacle avoidance in neuro-robotic systems.

**Approach:**

We presented an all-optical biological reservoir computing framework that leverages optogenetics and calcium imaging to precisely regulate and record neuronal activities. A closed-loop system was developed incorporating the FORCE learning algorithm, which guided a virtual car through obstacle avoidance tasks.

**Results:**

The system demonstrated high accuracy and efficiency in navigating obstacles, achieving optimal performance after ∼150  s of training. OS significantly improved the obstacle avoidance success rate, enhancing the system’s adaptability and accuracy.

**Conclusions:**

The results highlight the potential of optogenetically controlled biological neural networks in neuro-robotic systems, showcasing their capability to achieve accurate and efficient obstacle avoidance through physical reservoir computing.

## 1 Introduction

Biological intelligence excels in processing complex information with remarkable efficiency, achieving high data utilization and operating with minimal energy consumption.^1^^–^^3^ Due to their intrinsic complex dynamics and spatiotemporal processing capabilities, biological neural networks have recently gained popularity as reservoirs in physical reservoir computing (PRC),^4^^–^^6^ making them well-suited for performing multiple computational tasks. PRC leverages a reservoir to transform input data into a high-dimensional space, followed by a readout mechanism that extracts patterns from the system’s complex states. One of the key advantages of PRC is that the reservoir itself remains untrained, with only the readout weights requiring optimization. This greatly reduces the computational burden compared with traditional recurrent neural networks, which involve training both the network structures and weights, leading to shorter learning times and lower overall training costs.^7^^,^^8^ The inherent versatility and computational efficiency of PRC make it a promising approach for handling a wide range of real-world tasks, from image recognition to robotic control optimization.^5^

Biological neural networks have been effectively utilized as reservoirs in physical reservoir computing systems, where neural activity can be recorded through microelectrode arrays (MEA) to extract meaningful signals from the network.^9^^,^^10^ One of the most notable applications of this approach is the control of robotic cars navigating mazes, demonstrating the powerful capabilities of biological neural networks in executing complex pathfinding tasks.^11^ In another related study, biological neural networks were integrated into sensorimotor feedback loops to enable robots to perform obstacle avoidance maneuvers with remarkable efficiency.^10^ However, this method has its limitations, primarily because single electrodes in MEA setups often capture signals from multiple neurons, which reduces the spatial resolution of signal recordings.

Optical methods, such as calcium imaging, have been employed to record the fluorescence intensity of individual neurons over a wide field of view, thereby indirectly reflecting neuronal activity.^12^^–^^15^ The recent development of calcium fluorescence proteins, with their fluorescent half-rise time of under 50 milliseconds, has enabled the clear visualization of neural activity,^16^^,^^17^ significantly enhancing the applications of calcium imaging. Optogenetics, a method of noninvasive stimulation, involves the genetic modification of cultured neurons to express light-sensitive proteins, enabling the activation of neurons exclusively through light of a specific wavelength.^18^^,^^19^ This approach has garnered significant attention due to its ability to reduce neuronal damage.^20^^,^^21^ Optogenetics has been widely applied in various fields, from studying brain functions such as memory, learning, and behavior to enabling precise control of neural networks in bio-hybrid systems, such as controlling the movements of robotic systems or virtual environments.^22^^–^^25^ The combination of calcium imaging with optogenetics could facilitate the simultaneous monitoring and control of neural activity within biological neural networks,^26^^,^^27^ offering substantial value for the study of physical reservoir computing with *in vitro* neural networks.

In this paper, we propose a biological reservoir computing framework based on optogenetics and calcium imaging. This framework utilizes an *in vitro* neural network as a physical reservoir, where input signals are delivered to the network via optogenetic techniques, and the network’s activity is recorded through calcium imaging. We employ the first-order reduced and controlled error (FORCE) learning algorithm^11^^,^^28^^–^^30^ to facilitate the training process of the biological reservoir framework. To implement this system, we developed a setup capable of performing both patterned optogenetic stimulation (OS) and calcium imaging simultaneously. The optogenetic stimulation is rapidly reconfigurable, effectively supporting the selective stimulation of specific neurons in the experiment, whereas the high-spatial-resolution calcium imaging system accurately records the state of each reservoir node. Coupled with the FORCE learning algorithm, this enables fast and efficient online learning. The efficacy of this framework was demonstrated through its application in a virtual robotic obstacle avoidance task, where it was observed that the biological reservoir framework attained over 95% obstacle avoidance success using fluorescence signals from a mere 15 neurons after 150 s of training. This outcome serves to underscore the notion that patterned optical stimulation can effectively alter the states of reservoir nodes. Furthermore, an analysis of neural network properties both before and after the training period corroborated this finding. It was observed that improved training outcomes in achieving optimal performance from the biological reservoir can be attained through a pretrained biological reservoir, likely due to the enhanced plasticity of the biological neural network under optical stimulation. These observations highlight the potential merits of integrating optical methodologies with biological reservoirs, thereby offering novel insights into the domain of biological intelligence.

## 2 Experimental Setup and Method

### 2.1 Primary Neuron Culture

Cortical tissues were obtained from embryonic mice at embryonic day 15 (E15) from pregnant mice (SPF Beijing Biotechnology Co. Ltd., Beijing, China). To ensure tissue integrity and cell viability, all procedures were strictly performed on ice. After carefully isolating the cortical tissues, the tissues were cut into small pieces and then immersed in DMEM (Macgene, Beijing, China). The tissues were gently washed twice with DMEM, followed by digestion in 0.125% trypsin (Macgene) containing deoxyribonuclease (DNase I, Solarbio, Beijing, China) at 37°C for 20 min. After digestion, the tissue was further dissociated into a single-cell suspension by gentle pipetting. The cell suspension was collected and centrifuged at 80×g for 5 min to pellet the cells. After centrifugation, the supernatant was carefully discarded. To improve cell survival and optimize culture conditions, the cells were resuspended in Neurobasal Plus Medium (Gibco, Waltham, Massachusetts, United States) supplemented with B27 Plus (Gibco, Waltham, Massachusetts, United States), GlutaMax (Gibco, Waltham, Massachusetts, United States), and penicillin–streptomycin (Macgene). The resuspended cells were then seeded at a density of 3 to $4\times 10^{5}\;\text{cells}/\mathrm{ml}$ into 24-well plates (Corning, Corning, New York, United States) pre-coated with poly-D-lysine (PDL, Sigma-Aldrich, St. Louis, Missouri, United States).

The culture plates were incubated at 37°C in a humidified atmosphere with 5% ${\mathrm{CO}}_{2}$. On the third day *in vitro* (DIV3), cells were transduced with AAV2/9-CAG-CheRiff-eGFP-WPRE-bGHpA and AAV2/9-CAG-jRCaMP1b-WPRE-bGHpA. These adeno-associated viral vectors efficiently express the optogenetic actuator CheRiff^19^ and the calcium indicator jRCaMP1b.^16^ After ∼14 days of culture, the neurons were fully differentiated, and subsequent optogenetic stimulation and calcium imaging experiments were conducted.

### 2.2 Experimental Setup

**Fig. 1:**
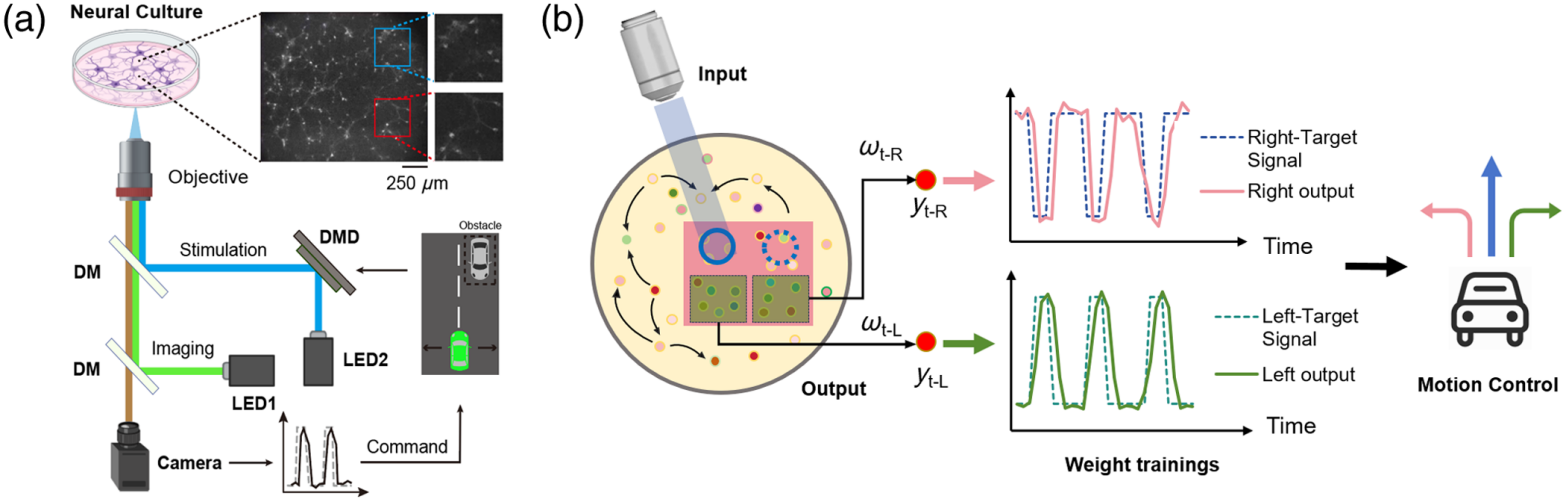
Physical reservoir computing based on *in vitro* neuronal culture. (a) Schematic of the experimental setup. Calcium imaging and optical stimulation for optogenetics control are performed using green light and blue light, respectively. DM, dichroic mirror; DMD, digital micromirror device; LED, light-emitting diode. (b) Schematic of the experimental principle. The pink rectangular area represents the neuron being imaged, and the two green rectangular regions inside indicate the left and right calculation execution areas, and the two blue circles denote the left and right stimulation perception areas.

Our experimental setup, depicted in Fig. 1(a), features a high-precision sCMOS camera that records the activity of cultured mouse cortical neurons in a petri dish, whereas optical stimulation is applied through a digital micromirror device (DMD). Neuronal firing leads to an influx of intracellular calcium ions, which interact with the fluorescent indicator jRCaMP1b,^16^ causing changes in fluorescence intensity. The green LED (LED1, with a center wavelength of 565 nm) serves as the excitation light source for calcium fluorescence imaging, forming a microscopy module in conjunction with the objective lens and camera, which is used to capture calcium fluorescence signals from the neuronal network. Light from the blue LED (LED2, center wavelength: 470 nm) is modulated by the DMD and focused onto the sample plane via a 4f system comprising a lens (focal length: 200 mm) and an objective lens (10× magnification). This setup is employed to provide optogenetic stimulation to the cultured neuronal network. Through the precise adjustments, the system can achieve an optical stimulation resolution of less than 2  μm, with the maximum optical stimulation frequency exceeding 1 kHz. In addition, when the neurons are activated and start firing, calcium ions enter the cells, causing changes in fluorescence, which could be captured by the sCMOS camera. The neuronal activity data are then used to control a virtual car, allowing it to navigate and avoid obstacles.

### 2.3 Strategy for Obstacle Avoidance Task

As shown in Fig. 1(b), we designated two “stimulation perception areas” and two “calculation execution areas” to manipulate the movement of the virtual car in the left and right lanes, respectively. Our closed-loop reservoir computing system comprises three main layers: an input layer, a reservoir layer, and an output layer. The input layer is responsible for mapping time series signals to the reservoir layer, which is achieved by directing blue light onto specific regions within the neuronal network—referred to as the left and right stimulus perception areas—via the DMD. We employed a ring-shaped stimulation pattern due to several key advantages. First, we experimentally found that network activity is more effectively controlled by the size of the area enclosed by the light stimulus, rather than the total illuminated area itself (Video 1). A ring with a large diameter can therefore elicit widespread network activity more efficiently than a solid circle of equivalent area. Second, this geometry minimizes potential phototoxicity by reducing direct, high-intensity light exposure to the dense core of the neuronal cluster.^31^ The optical power density of the stimulation spot was set to $∼1\;\mathrm{mW}/mm^{2}$, a level chosen to ensure robust neuronal activation. Finally, to encode spatial information, stimuli corresponding to obstacles in the left and right lanes were delivered to two distinct “stimulation perception areas” in the upper-left and upper-right regions of the culture. To ensure reliable signal propagation to the reservoir layer, each of these areas was selected to encompass over 20 neurons. Stimulation was triggered whenever the vehicle-to-obstacle distance dropped below a predefined threshold and was terminated once the vehicle either completed a lane change or moved past the obstacle (Video 2). The reservoir layer operates with random weights that facilitate nonlinear interactions among neurons, allowing the input data to be dynamically expanded and transformed over time. This nonlinear dynamic response generates a rich, high-dimensional representation of the input signals. Within this reservoir, ∼20 neurons from each of the left and right calculation execution areas are selected as the execution units. These neurons, which are loaded with fluorescent indicators, exhibit changes in fluorescence intensity due to calcium ion influx during neuronal firing. By capturing these changes in brightness using fluorescence imaging, the neuronal activity is recorded. The output layer then acts as a linear mapping that translates the high-dimensional state vectors from the reservoir layer into the desired output values. During training, the weights of the output layer are adjusted to minimize the difference between the generated output and the target, ensuring close alignment.

In our system, obstacle avoidance for a virtual car is achieved by controlling its movement direction using the output signal from the left and right calculation execution areas. Specifically, when an obstacle is detected on the left side, the neurons in the left sensory region generate an output signal to prompt the car to turn right. The car only responds when the wave signal reaches a certain threshold. In detail, the threshold is defined as half of the maximum value of the target signal pulse. A similar mechanism controls right-side obstacle detection and leftward movement (Video 2).

### 2.4 Neural Network Analysis

The spike template-based method was employed for detecting spike events, as implemented in FluoroSNNAP.^32^ Cross-correlation, a widely used technique to assess the relationship among time series, was applied to analyze neuronal signals.^33^ After detecting spike events, the extracted events were converted into simulated calcium fluorescence signal curves. The correlation among the simulated calcium signals of different neurons was then calculated. The connectivity between neuron i and neuron j was defined based on the following procedure. To calculate the connectivity, the timing of events for neuron j was shuffled to create surrogate event data, ensuring that the number of events in the surrogate sequence remained consistent with the original sequence.^34^ The correlation coefficient between the event sequence of neuron i and the surrogate event sequence of neuron j was then computed. This process was repeated N times, generating a series of surrogate correlation coefficients, where N=500 in this study. The connectivity between neuron i and neuron j is defined as $A_{ij}$. If the correlation coefficient $CC_{ij}$ exceeds 99% of the values in the surrogate sequence $A_{ij}=1$, a functional connection is formed between neuron i and neuron j. By contrast, if $A_{ij}=0$, no connection is constructed. To build an undirected functional connectivity graph, symmetry of the adjacency matrix was imposed, ensuring that $A_{ij}=A_{ji}$ for all pairs of neurons. Once the functional connections of the neural network were determined, global efficiency (GE), modularity (Q), clustering coefficient (*Coef*), and network density (*Nd*) were calculated^35^^–^^37^

$$GE=\frac{1}{N(N-1)}\underset{i\neq j\in G}{\sum }\frac{1}{d_{ij}},$$ (1)

$$Q=\frac{1}{2m}\underset{i,j}{\sum }(A_{ij}-\frac{k_{i}k_{j}}{2m})\delta (c_{i},c_{j}),$$ (2)

where G is the set of neuron nodes, $d_{ij}$ is the shortest path length between neuron node i and network node j, and N is the total number of nodes. We note that $d_{ij}=\infty$ for disconnected pairs (i,j), and $A_{ij}$ is the connection between node i and node j, as described above. $k_{i}$ and $k_{j}$ represent the degrees of node i and j respectively, and m is the total number of edges in the network. These community assignments are determined by applying a community detection algorithm that iteratively optimizes the modularity to find the partition that yields the maximum modularity value. Specifically, our analysis employed the Louvain method^38^ as implemented in the Brain Connectivity Toolbox.^39^ $c_{i}$ is the community to which node i is assigned. $\delta (c_{i},c_{j})$ takes the value of 1 when node i and node j belong to the same module and 0 otherwise. The clustering coefficient is used to measure the degree to which nodes in a graph tend to cluster together. For each node v, the local clustering coefficient C(v) is defined as

$$C(v)=\frac{2E(v)}{k_{v}(k_{v}-1)},$$ (3)

where E(v) represents the number of edges among the neighbors of node v and $k_{v}$ is the degree of node v (i.e., the number of connections to other nodes). The global clustering coefficient *Coef* is calculated as the average of the local clustering coefficients across all nodes in the network

$$Coef=\frac{1}{N}\underset{v\in V}{\sum }C(v),$$ (4)

where N is the total number of nodes in the network. The density of the network, *Nd*, quantifies the proportion of possible connections that are actual connections. It is calculated using the equation

$$Nd=\frac{2E}{N(N-1)},$$ (5)

where E is the total number of edges in the network and N is the total number of nodes. This measure provides insight into the sparsity or richness of connections within the network.

To highlight the more important nodes within the neural network, we implemented the following visualization procedure. For each neuron i, we identified the p neurons with the highest pairwise correlation coefficients, denoted as $CC_{ij}$. Directed connections were then established from neuron i to these p, most strongly correlated neurons. This process resulted in a directed network where a connection from neuron i to neuron j is denoted as $l_{ij}$. Subsequently, we quantified the importance of each neuron by calculating its in-degree, which represents the total number of incoming connections it received.^40^

### 2.5 Low Computational Cost Cell Detection (LCCD) for Neuron Identification

A method called LCCD is used to identify neurons in the calculation execution areas,^41^ followed by manual supplementation to ensure accuracy. LCCD is an efficient cell detection method based on filtering and thresholding, designed for processing large-scale calcium imaging data. LCCD segments the data into multiple short time frames and applies moving average filters to eliminate slow temporal trends and imaging noise. The method identifies individual neurons by detecting the maximum intensity of the filtered data at each pixel location, enhancing contrast through contrast-limited adaptive histogram equalization,^42^ and employing the Otsu method^43^ for binarization. Closed regions representing neurons are detected and filtered based on their elliptical shape using metrics of eccentricity and area ratio. The regions of interest (ROI) detected in individual frames are consolidated by removing overlapping or nonneuron-sized ROIs. An ROI is a specific area within an image selected for detailed analysis, in this case, representing individual neurons. This approach effectively isolates single neurons in high-density and overlapping cell environments, demonstrating high detection accuracy and computational efficiency when processing large-scale imaging data from modern wide-field microscopes.

### 2.6 Echo State Property Assessment

To investigate the echo state property (ESP) of our neuronal cultures, we assessed both the reproducibility of their responses to identical stimuli and their short-term memory capacity. The cultures were driven by an independent and identically distributed random binary input sequence v(t)∈{0,1}, over four repeated 120-s trials, with a 5-min rest interval among each trial. A value of v(t)=1, triggered a 500 ms rectangular light pulse (200 ms illumination, and 300 ms dark) delivered via the DMD, whereas no stimulus was applied for v(t)=0; the resulting relative fluorescence change (ΔF/F) of individual neurons,^31^ denoted as $x_{n}^{i}(t)$ for neuron n in trial i, was recorded at 10 Hz and subsequently denoised^44^ prior to further analysis.

A core tenet of the ESP is that the reservoir’s response should be uniquely determined by the input,^45^ making it reproducible across trials.^46^^,^^47^ To quantify this, we performed a dynamic cross-correlation analysis using a sliding window approach. Each 120-s recording was segmented into 30-s windows ($L_{w}=300$ points) with a 1-s step size ($L_{s}=10$ points). For each window h, we computed the normalized cross-correlation function, $C_{ij,h}^{n}(l)$, between corresponding segments from two trials (i and j), where l is the time lag. From this function, we extracted two time-evolving metrics: the maximum cross-correlation coefficient, ${\mathrm{MaxCorr}}_{ij}^{n}(h)=\underset{l}{\max}\;C_{ij,h}^{n}(l)$ to measure response similarity, and the time lag $d_{ij}^{n}(h)=\arg\;\underset{l}{\max}\;C_{ij,h}^{n}(l)$, to measure the temporal phase shift. This allowed us to dynamically track the stability and consistency of neural activity patterns.

A functional reservoir must also possess short-term memory, which we quantified using the memory capacity (MC) metric.^48^^–^^50^ This involved training a linear readout to reconstruct past input values, v(t−τ), from the current reservoir state, x(t), which was derived from the neuronal activity signals. For each time delay τ, a unique weight vector $W_{\tau }$ was computed via ridge regression^45^ to produce an output y^τ(t)=Wτ·x(t). The weights were determined by the closed-form solution $W_{\tau }=Y_{\tau }^{T}X(X^{T}\;X+\lambda I{)}^{-1}$, where X is the matrix of reservoir states and $Y_{\tau }$ is the vector of target historical inputs. The regularization coefficient λ was set to 0.01. The reconstruction accuracy for each delay is given by the memory function $f_{\tau }$

fτ=(∑t(v(t−τ)−v¯)(y^τ(t)−y^τ¯))2∑t(v(t)−v¯)2·∑t(y^τ(t)−y^τ¯)2, (6)

where $\overset{¯}{v}$ and y^τ¯ are the temporal means of the input and output sequences, respectively. Finally, the total MC is defined as the sum of the memory functions over all delays τ

$$\mathrm{MC}=\overset{\infty }{\underset{\tau =1}{\sum }}f_{\tau }.$$ (7)

### 2.7 Weight Adjustment in Closed-Loop Reservoir Computing System

For post-screening, weight vectors were initialized using normal and Bernoulli distributions to ensure the initial network balance. For each weight ω of a neuron, a random number is first drawn from a standard normal distribution (mean of “0” and standard deviation of “1”) and then divided by the normalization factor S, defined by the expression

$$S=\sqrt{pz\times N},$$ (8)

where pz denotes the probability of nonzero weights and N represents the number of neurons. The Bernoulli distribution is subsequently utilized to determine whether a weight should be set to 0, thereby controlling the sparsity of the weight vector. Adjusting the parameter pz allows for the management of the proportion of nonzero elements. Weights generated using the normal distribution exhibit uniform randomness, aiding in maintaining the initial balance of the network. Initializing the weights with a mean of 0 ensures that the network does not exhibit any initial directional bias. The normalization factor S adjusts the range of initial weights, preventing issues such as vanishing or exploding gradients, thus improving the training efficiency and performance of the network.

During the training process of our experiments, FORCE learning is applied to dynamically adjust the weights of the connections between the reservoir layer and the output layer. FORCE learning does not focus on suppressing errors; instead, it allows the output error to remain small enough to feed back into the network without disrupting the learning process. This method significantly reduces output error quickly and efficiently through rapid weight adjustments. It maintains a small error during the search process and eventually identifies a fixed set of readout weights that can sustain minimal error without further modifications. At each time interval Δt, the deviation of the network output from the target function is calculated, and the readout weights are adjusted accordingly. Δt represents the time interval for weight updates, whereas the basic integration time of the network can be shorter than Δt. At time t, the training process reads out the current network FORCE output as

$$w^{T}(t-\Delta t)r(t),$$ (9)

where r(t) denotes the firing rate of neurons at time t and $w^{T}$ denotes the transpose of the weight matrix. The error between this output and the target output is defined as

$$e_{-}(t)=w^{T}(t-\Delta t)r(t)-f(t),$$ (10)

where f(t) represents the target signal generated at time t based on the environment of the car. FORCE learning uses the recursive least squares method to update the weights

$$w(t)=w(t-\Delta t)-e_{-}(t)P(t)r(t),$$ (11)

where (P(t)) is the inverse of the covariance matrix, which is updated simultaneously with the weights. The update rule for (P(t)) is

$$P(t)=P(t-\Delta t)-\frac{P(t-\Delta t)r(t)r^{T}(t)P(t-\Delta t)}{1+r^{T}(t)P(t-\Delta t)r(t)}.$$ (12)

## 3 Results and Discussion

### 3.1 Calcium Imaging and Optogenetic Stimulation of the Biological Neural Network

**Fig. 2:**
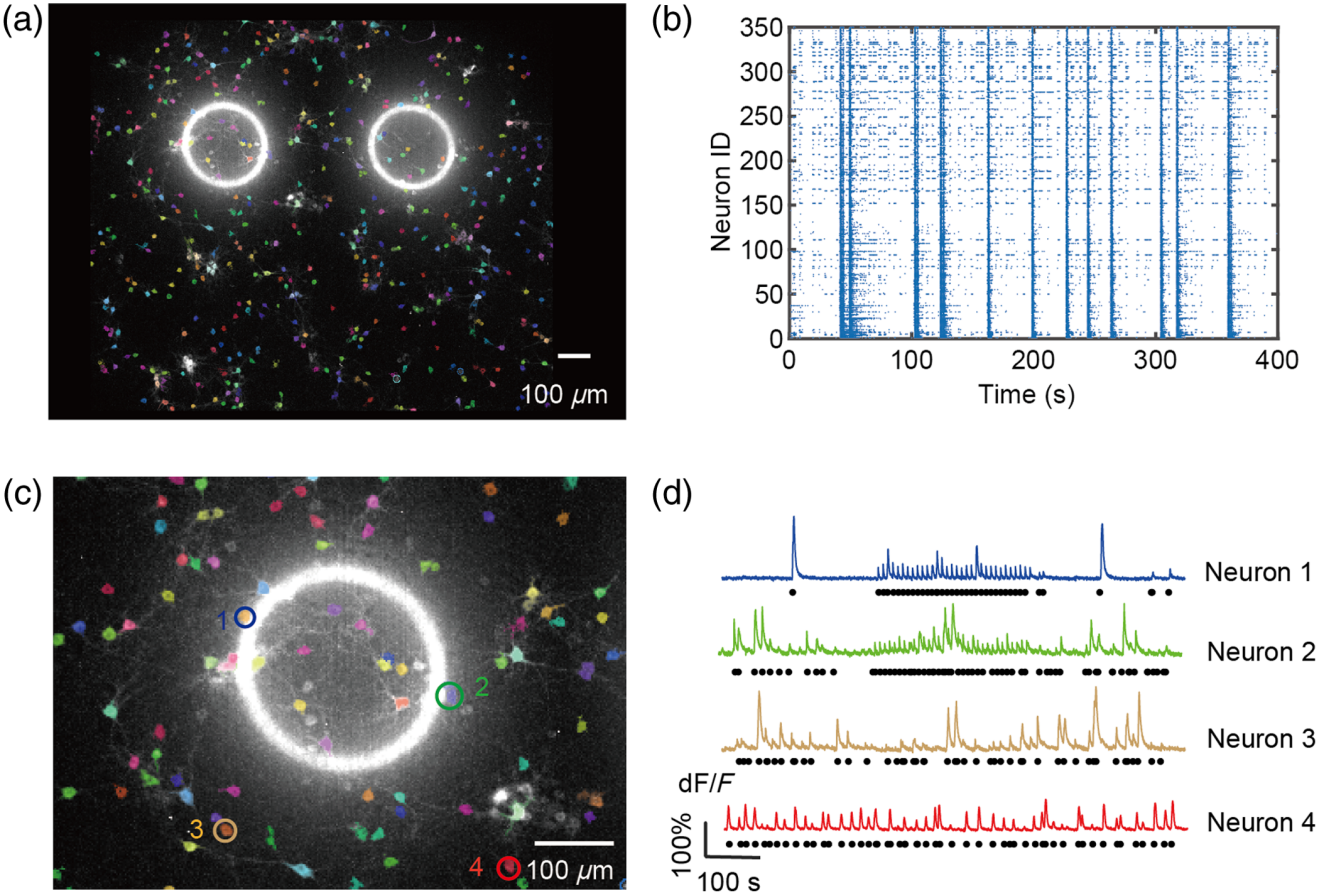
Calcium imaging and optogenetic stimulation of the biological neural network. (a) Maximum brightness overlay of calcium imaging, displaying the calcium ion activity of the neural network. Two bright circles indicate the areas where optogenetic stimulation was applied. (b) Spontaneous calcium fluorescence activity of 350 neurons in the field of view over a 400-s period. (c) A magnified view of panel (a), highlighting four neurons: two located within the optogenetic stimulation rings (blue and green circles) and two farther away from the stimulation regions (yellow and red circles). (d) Calcium fluorescence changes in the four selected neurons before, during, and after optogenetic stimulation. The orange rectangular marks the time period when the optogenetic stimulation was applied. Each black dot corresponds to a spike event, detected based on peak changes in the fluorescence intensity signal.^32^

The experiment was conducted on the 15th day of the growth of the biological neural network. Figure 2(a) shows a calcium imaging map of the neural network, specifically a maximum brightness overlay image of the neurons after neuronal image segmentation. This image captures the calcium ion activity of 350 distinct neurons, with the two circular regions representing the areas where optogenetic stimulation was applied. The bright rings in the image mark the precise locations where blue light was introduced to stimulate the neurons in the left and right regions of the network.

Figure 2(b) depicts the spontaneous firing activity of the 350 neurons over a 400-s period. The data reveal that a majority of neurons in the network exhibit vigorous calcium activity throughout the 400 s. The network demonstrates synchronous firing behavior, with stable and consistent neural activity, indicating that the biological neural network has reached a mature stage of growth. This maturity is crucial for ensuring that the network can support the subsequent obstacle avoidance tasks performed by the virtual car.

Figure 2(c) is a magnified view of Fig. 2(a), focusing on a smaller subset of the neural network. Four specific neurons were selected for further observation: two neurons located within the optogenetic stimulation rings, and two neurons positioned farther away from the stimulation regions. By analyzing the calcium fluorescence changes in these neurons before and after optogenetic stimulation, we can assess their responsiveness to the applied light stimuli. In addition, Fig. 2(d) shows the activity changes in the four selected neurons, presenting their calcium fluorescence signals before, during, and after optogenetic stimulation. The neurons situated within the stimulation circles exhibit a strong response to the blue light, with a significant increase in neural activity observed during the application of the light. Once the stimulation is removed, the neurons’ activity quickly returns to baseline levels. On the other hand, the neurons farther away from the stimulation area show no change in activity. These results demonstrate the network’s robust responsiveness to optogenetic stimulation and its ability to recover rapidly after the stimulus is withdrawn, confirming the reliability and effectiveness of this biological neural network for the planned tasks.

### 3.2 Impact of Optogenetic Stimulation on Neural Network Characteristics and Connectivity

**Fig. 3:**
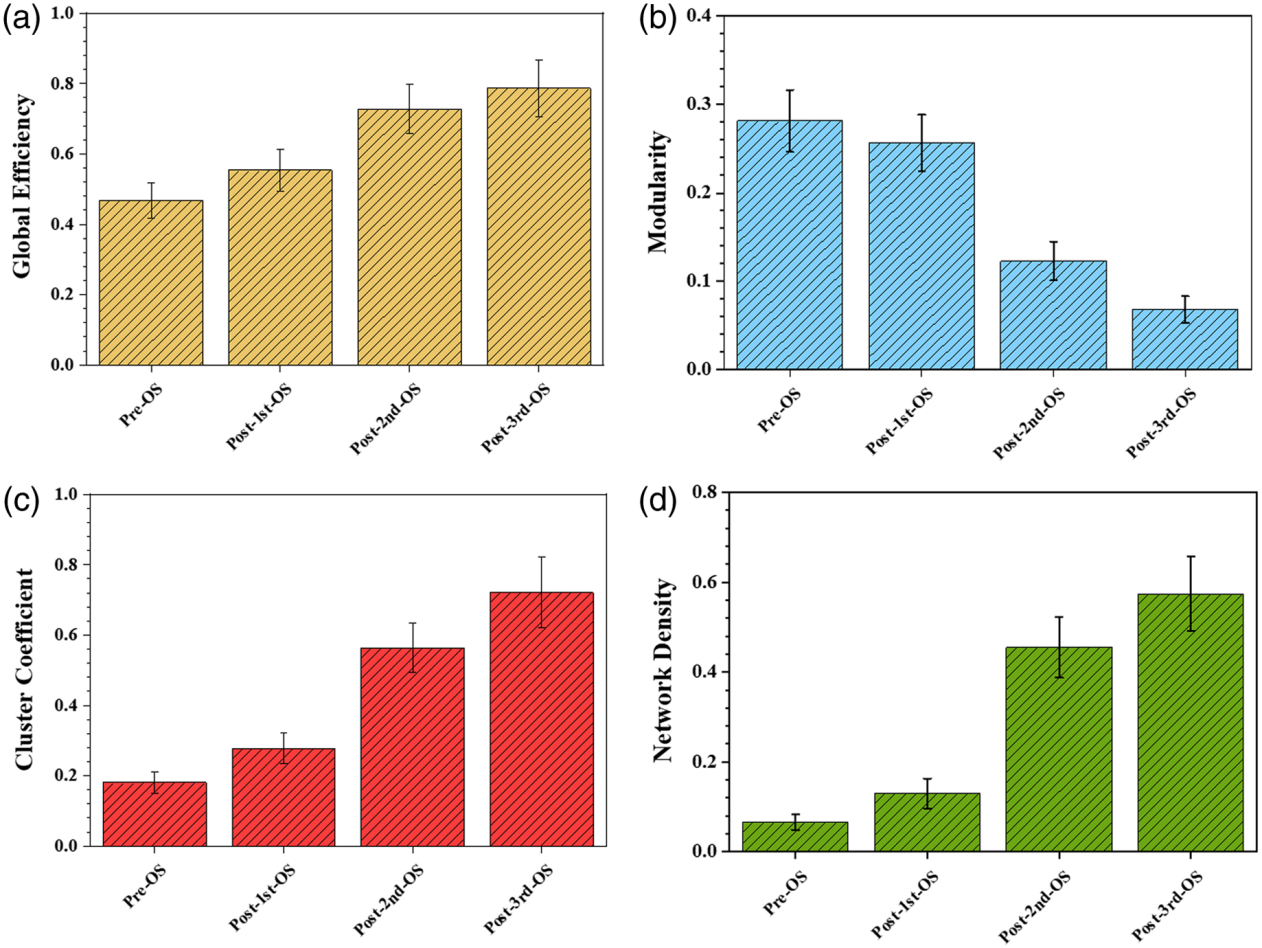
The four bar charts show the effects of OS on neural network characteristics. (a) Global efficiency increases after each stimulation, indicating better information flow; (b) modularity decreases slightly after the first stimulation and continues to decline with further stimulations; (c) clustering coefficient rises, showing more local connections among neurons; (d) network density increases, indicating more overall connections in the network. Each bar represents different time points: before stimulation, and after the first, second, and third stimulations. Error bars indicate standard deviations.

To investigate the impact of OS on network topology, we analyzed network dynamics before and after stimulation during the closed-loop virtual vehicle obstacle avoidance task (described in Sec. 3.4). The stimulation induced significant changes in several key network metrics, including global efficiency, modularity, clustering coefficient, and network density. As shown in Fig. 3(a), before stimulation, the global efficiency was relatively low, indicating suboptimal information transfer across the network. However, after the first stimulation, global efficiency showed a marked increase, which continued to rise with subsequent stimulations. This suggests that OS enhances the network’s ability to transmit information, making the network more efficient as stimulations progress. Modularity, reflecting the presence of distinct functional modules within the network, was initially high before stimulation, indicating well-defined functional divisions. Figure 3(b) shows that following the first stimulation, modularity slightly decreased, implying that OS disrupted some module boundaries and created a more integrated network structure. After the second and third stimulations, modularity continued to decrease, suggesting that the network progressively adjusted to a more cohesive structure, with functional modules becoming increasingly integrated over time. The clustering coefficient, which indicates the degree of local interconnectedness among neurons, was low before OS. It increased significantly after the first stimulation and continued to rise with further stimulations [Fig. 3(c)], highlighting that OS promotes the formation of tightly connected local neuron groups. This suggests that repeated OS strengthens local neural processing by fostering more robust cluster structures within the network. In addition, Fig. 3(d) displays that the network density, initially low before stimulation, increased significantly following each round of OS, showing that the stimulation not only reinforced existing connections but also facilitated the formation of new connections among neurons. This progressive increase in density indicates that OS leads to a more densely connected and highly integrated neural network.

We believe that the optogenetic stimulation surely enhances multiple aspects of neural network structure and function, improving global efficiency, local connectivity, and overall network integration. The effects of OS are especially evident in the progressive enhancement of global efficiency and clustering, with repeated stimulations further solidifying these improvements. Modularity initially decreases after the first stimulation and continues to decline with subsequent stimulations, indicating that OS promotes a gradual reorganization toward a more integrated network structure. This ongoing reduction in modularity suggests that the network is increasingly adjusting to a unified functional architecture as it responds to OS. These findings demonstrate the potential of OS as a tool for modulating and optimizing neural network characteristics, with implications for therapeutic interventions and the development of more effective artificial neural network models in computational neuroscience.

**Fig. 4:**
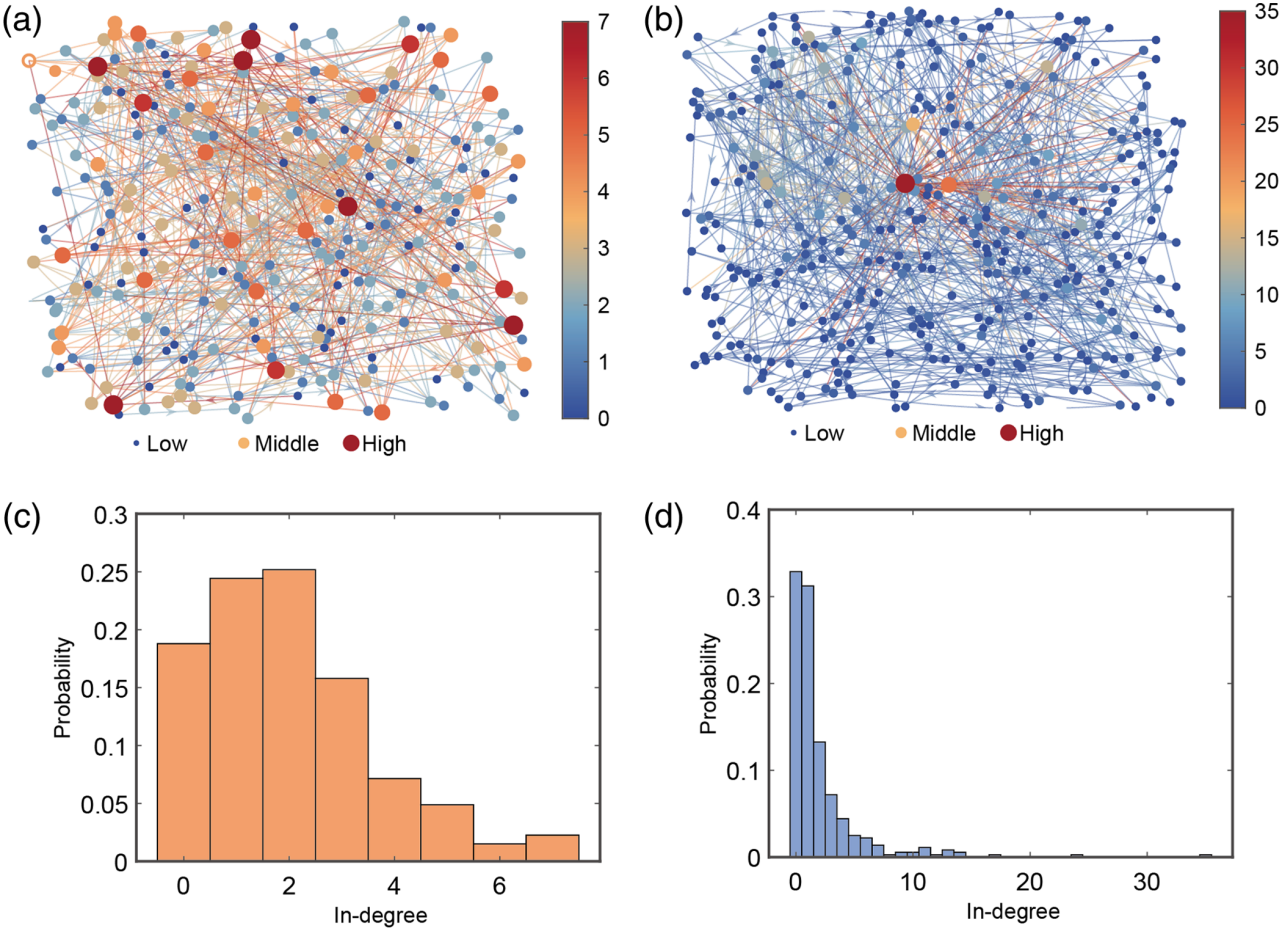
Impact of optogenetic stimulation on network connectivity and in-degree distribution. Connectivity maps shown in panels (a) and (b) were constructed by connecting each neuron to its two most strongly correlated neighbors (p=2). Node color corresponds to the in-degree, with warmer colors (e.g., red) indicating a higher number of incoming connections. (a) The network topology prior to optogenetic stimulation, characterized by a predominance of nodes with low-to-medium in-degree. (b) The network topology following stimulation, revealing a marked increase in high-degree nodes and strong connections (red nodes and lines). (c) The in-degree distribution before stimulation, showing that most neurons have a low number of incoming connections. (d) The in-degree distribution after stimulation, which exhibits a right-skewed tail, indicating the emergence of highly connected hub neurons within the network.

Furthermore, the impact of optogenetic stimulation on the connectivity of the neural network was investigated. Figure 4(a) shows the connectivity of the neural network prior to optogenetic stimulation. For better visualization, each node is connected to its two most correlated neurons (i.e., p=2), and the in-degree of a node, which is defined as its total number of incoming connections, serves as a proxy for its network importance.^40^ In this state, high-degree nodes (represented by darker red colors) and their corresponding high-intensity connections are relatively sparse, and the overall network exhibits more evenly distributed connection strengths, with most connections falling into the medium-intensity range (orange nodes and lines). This suggests that, in the absence of external stimulation, the neural network’s connections are primarily of moderate and lower strength, with fewer regions of high activity. By contrast, Fig. 4(b) illustrates the neural network connectivity after optogenetic stimulation. Compared with the pre-stimulation state, there is a noticeable increase in high-intensity connections, as indicated by the larger number of red and orange nodes and lines. Several central nodes (in red) now exhibit significantly stronger connections to other neurons, indicating that these nodes have become more active and potentially more influential in processing information within the network. To validate this observation, we also analyzed the connectivity when each neuron was connected to its single most correlated neighbor (p=1) and to its top three and four neighbors (p=3, 4) and found that the degree distribution showed a consistent trend (Fig. S1 in the Supplementary Material). In addition, we examined the network connectivity and node degree distribution of the original, undirected graph, which similarly revealed that specific nodes were significantly enhanced after stimulation (Fig. S2 in the Supplementary Material). The increase in connection strength likely reflects the network’s response to external stimulation, where certain neurons become highly active and the overall network enters a heightened state of activity.

The in-degree distribution of the neural network before and after optogenetic stimulation is also calculated. Figure 4(c) represents the degree distribution prior to stimulation. As can be seen, most nodes have low in-degrees, with the majority of the connections concentrated in the lower range, indicating that a large portion of the neurons in the network are involved in fewer interactions. Figure 4(d) shows the in-degree distribution after optogenetic stimulation. The distribution exhibits a significant shift, with a broader range of node degrees and a higher occurrence of neurons with a large number of connections. This suggests that optogenetic stimulation leads to an increase in the connectivity of certain neurons, driving the formation of highly connected hub nodes within the network.

The analysis of the neural network’s connectivity before and after optogenetic stimulation reveals a significant transformation in its structure and dynamics. Prior to stimulation, the network shows relatively balanced, moderate-strength connections, with fewer high-activity nodes. However, after optogenetic stimulation, there is a marked increase in the number of highly connected neurons, with certain nodes becoming central hubs, demonstrating enhanced activity and influence within the network. The in-degree distribution further supports this observation, showing a broader and more diversified range of connections post-stimulation. These findings suggest that optogenetic stimulation not only amplifies neural activity but also enhances the network’s connectivity, potentially improving its computational capacity and ability to process complex tasks.

### 3.3 Echo State Property of Cultured Neuronal Network

**Fig. 5:**
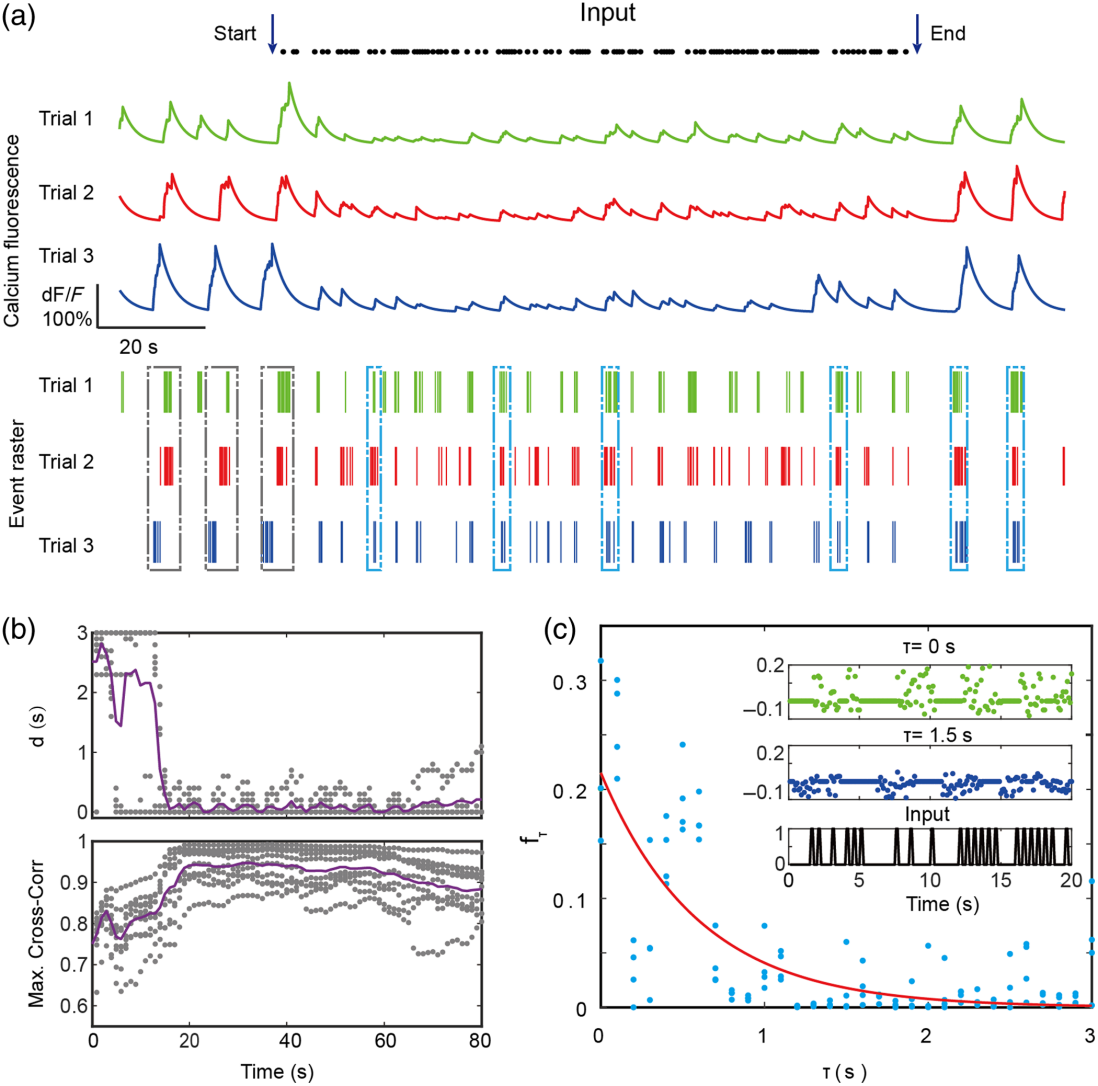
Assessment of the ESP in the neuronal culture. (a) Denoised calcium fluorescence traces (top panel) and corresponding event rasters (bottom panel)^44^ for a single representative neuron across three trials subjected to an identical input stimulus (black dots). Dashed gray boxes highlight early time periods where the neuron’s firing events are asynchronous across trials. Conversely, dashed blue boxes indicate later periods where the events become coherent and synchronized by the input. (b) Temporal evolution of the absolute time delay, d (top panel), and the maximum cross-correlation coefficient (bottom panel) among the calcium traces of trial 1 and trial 3. Gray dots represent the values calculated for individual neurons, whereas the solid purple line indicates the average across a cohort of 10 neurons. The decrease in time delay and increase in correlation over time demonstrate the convergence of network states. (c) The memory function, $f_{\tau }$, plotted against the time delay, τ, to quantify the network’s short-term memory. Data points (blue dots) are from four independent experiments. The red line represents an exponential fit to the data. Inset: The predicted output, y^τ(t), for delays of τ=0  s (top) and τ=1.5  s (middle), shown with the original input signal (bottom) for comparison.

To utilize the biological neuronal network (BNN) as a physical reservoir, it is essential to first confirm that it possesses the ESP. We therefore conducted an evaluation of the ESP in our neuronal cultures. To this end, an identical input sequence was used to stimulate the neuronal culture three consecutive times [Fig. 5(a)]. We then analyzed the resulting fluorescence signals from the same representative neuron across these three trials. The similarity and temporal “distance” among pairs of calcium fluorescence traces were quantified by calculating their maximum cross-correlation coefficient and the absolute value of the corresponding time delay within a 30-s sliding window (see Sec. 2.6).

Initially, the calcium signals from the same neuron across the three trials exhibited a significant phase difference. However, upon the introduction of the identical stimulus sequence, this phase difference progressively diminished, eventually stabilizing near 0 [Fig. 5(b)]. Concurrently, the maximum cross-correlation coefficient increased, stabilizing at a high value of ∼0.95. This demonstrates that the initial state of the network only transiently affects its subsequent dynamics, confirming that the neuronal culture possesses the ESP.

Furthermore, we quantified the network’s MC. We selected a 2-min segment of the relative calcium fluorescence intensity during the input period to serve as the system’s state trajectory. We then calculated the memory function $f_{\tau }$ and observed that $f_{\tau }$ rapidly decayed for delays τ>1  s [Fig. 5(c)]. An exponential fit applied to the memory coefficients obtained from four independent experiments yielded a characteristic decay constant of ∼0.60  s and MC≈0.13. This finding reveals that BNNs have a short-term memory lasting several hundred milliseconds, which is consistent with previous studies,^6^^,^^51^ rendering them effective as reservoirs for processing time series data with a ∼1  s timescale.

### 3.4 Closed-Loop Reservoir Computing System for Obstacle Avoidance

The FORCE learning algorithm^28^ is employed to dynamically adjust the neuronal weights in the system. This algorithm allows for rapid and precise weight updates to minimize error continuously, helping to determine a set of fixed readout weights that ensure stable performance. It is important to note that the target signal governing the virtual car’s movement is not a regular square wave; instead, it peaks when an obstacle is detected on one side and remains at a trough during normal driving. The outputs from the two distinct calculation execution areas control the left and right turns of the virtual car, ensuring smooth and accurate navigation around obstacles.

**Fig. 6:**
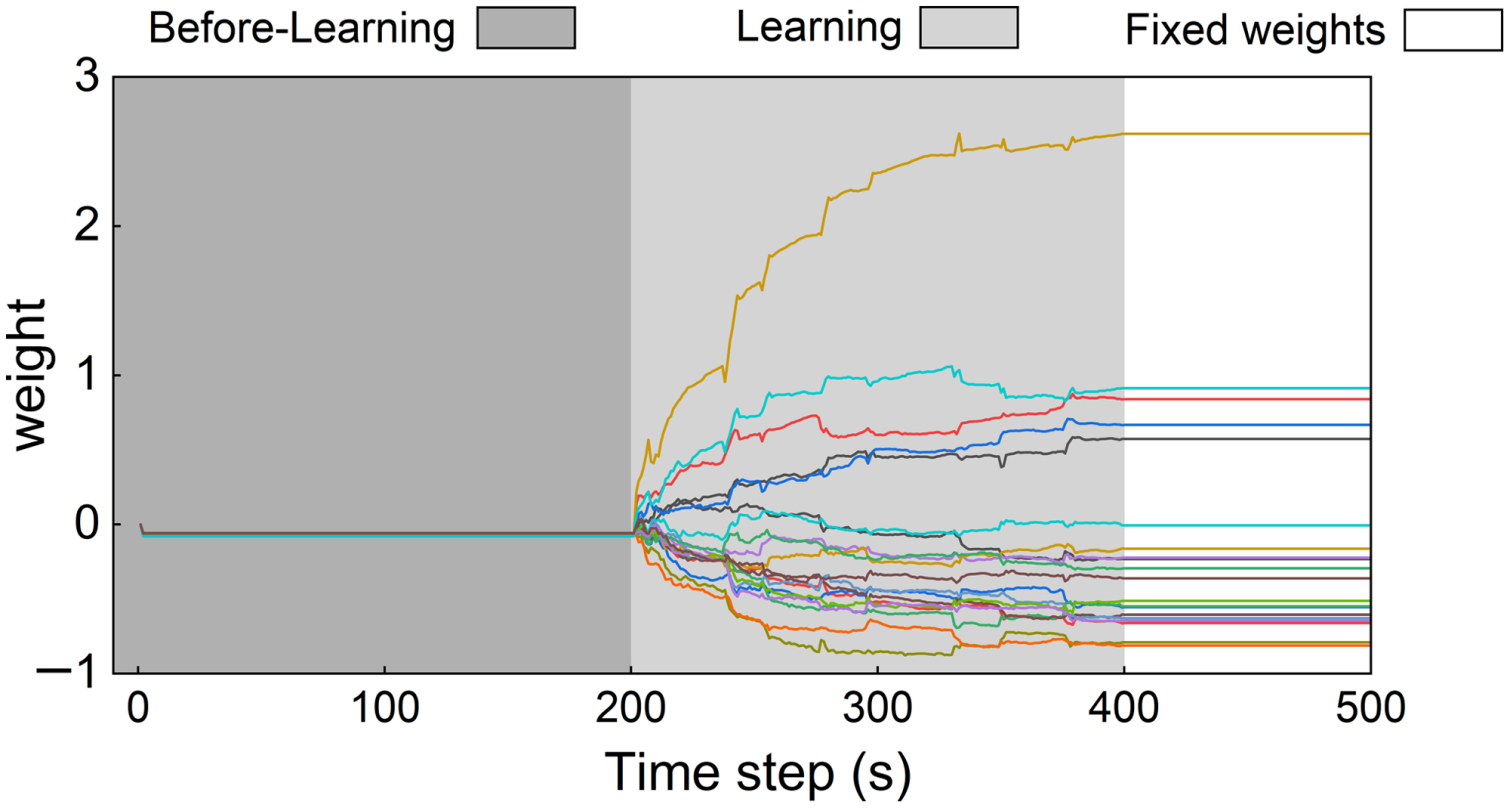
Weight adjustment during the learning process using the FORCE method. The graph shows the evolution of synaptic weights over 500 s, divided into three phases: before-learning (shaded dark gray), learning (light gray), and fixed weights (white).

Figure 6 illustrates the progression of the learning process over a 400-s period, with a focus on synaptic weight adjustments using the FORCE method. In the initial phase (before-learning, shaded in dark gray), the synaptic weights remain stable and unaltered. As the learning process begins (learning, light gray area), the FORCE learning algorithm quickly adjusts the synaptic weights, resulting in a significant reduction in the error between the predicted and target signals. During this period, the weights are dynamically fine-tuned in response to fluctuations in the target signal, ensuring that the model closely tracks the desired output. As the learning process progresses, the weight adjustments become increasingly minor, signaling that the model is close to stabilization. By the time the learning phase concludes, around the 400-s mark, the weights have effectively converged to stable values, indicating that further adjustments are no longer necessary. In the final phase (fixed weights, white background), the synaptic weights remain constant, showing no further changes after the learning process has completed.

**Fig. 7:**
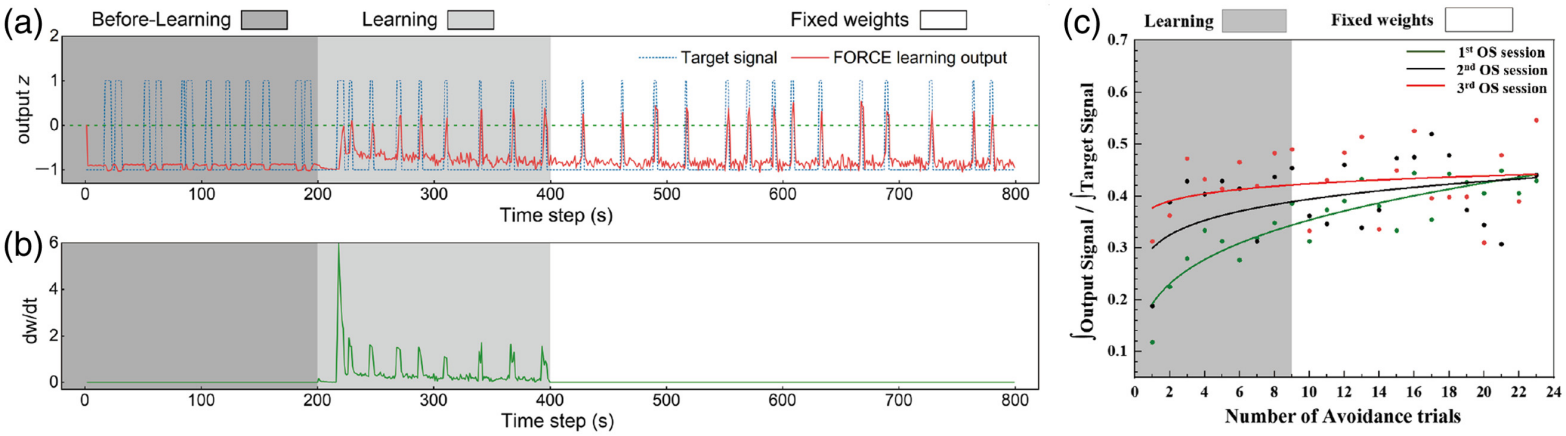
Output signal and weight adjustment during the learning process using the FORCE method. (a) The top panel shows the output signal (red line) and target signal (blue dashed line) over an 800-s period. During the before-learning phase (dark gray background), the system output deviates significantly from the target signal. During the learning phase (light gray), the output gradually aligns with the target signal, and in the fixed weights phase (white), the output closely follows the target with minimal error. (b) The bottom panel shows the rate of weight change (dw/dt) during the same period. In the before-learning phase, no weight adjustments occur. During the learning phase, significant weight changes are observed, which stabilize as the system approaches the fixed weights phase. (c) The proportion of the FORCE learning output integral relative to the target signal integral over the number of avoidance trials. Points represent experimental results, and the solid lines are fitted curves using a power function ($y=a\cdot x^{b}$).

Figure 7(a) presents the results from the left calculation execution area across an 800-s experimental timeline. In the before-learning phase (shaded in dark gray), no training of the synaptic weights occurs, which results in the actual output remaining unchanged and significantly different from the intended target signal. This lack of weight training prevents the system from executing accurate steering maneuvers, and the output (represented by the red line) fails to follow the target signal (depicted by the blue dashed line). As the system enters the learning phase (light gray), artificial synaptic weight training begins. Initially, there is a substantial discrepancy between the learning output and the desired target signal. However, with continuous adjustments, the system gradually learns to align its output with the target signal. This is reflected in the red output line progressively moving closer to the blue target signal line, which indicates improvement in the steering performance of the system. Over time, the FORCE learning algorithm fine-tunes the synaptic weights, reducing the error between the predicted output and the target. After ∼100  s of training, the system’s output begins to closely match the desired steering target, represented visually by the green dashed line in the graph (used to define the threshold). As seen in the final fixed weights phase (white background), the synaptic weights stabilize, and the output signal continues to follow the target signal closely, with minimal error. The system has effectively learned the necessary adjustments for accurate steering, as demonstrated by the improved alignment between the FORCE learning output and the target signal.

In Fig. 7(b), the rate of weight adjustments (dw/dt) for all neurons in the left calculation execution area is shown over an 800-s timeline. During the before-learning phase (dark gray), no weight changes occur. As the system enters the learning phase (light gray), significant weight adjustments are made, particularly in response to rises and descents in the target signal. These adjustments are crucial for minimizing the residual error between the neural network’s output and the target. Spikes in dw/dt reflect moments of rapid adaptation, ensuring the system aligns its output closer to the target. Over time, the frequency and magnitude of adjustments decrease, signaling improved prediction accuracy. By ∼400  s, the adjustments stabilize, indicating that the system has effectively learned the task and fine-tuned its performance, with minimal further changes in weights.

Figure 7(c) illustrates the proportion of the integral of the FORCE learning output curve relative to the target signal integral across avoidance trials in three optogenetic stimulation sessions, highlighting the impact of FORCE learning on system performance. During the learning phase (gray background), all three OS sessions show a gradual increase in this proportion, indicating improved alignment between the output and target signals as the number of trials increases. The third OS session notably begins with a higher initial proportion, indicative of the cumulative facilitative effects of repeated OS on the neural network’s functional plasticity and computational capacity. This underscores the ability of optogenetic stimulation to optimize neural dynamics and promote efficient information processing. In the fixed weights phase (white background), all OS sessions converge to a similar final proportion, reflecting the stabilization of system output—a hallmark of reservoir computing systems where the reservoir state remains fixed, whereas the readout weights encode task-specific information. These results underscore the beneficial impact of optogenetic stimulation in shaping neural dynamics and optimizing system performance.

The beneficial effects of OS observed in the FORCE learning performance [Fig. 7(c)] are consistent with earlier results demonstrating how OS alters network characteristics (Fig. 3) and connectivity patterns (Fig. 4). As shown in Fig. 3, repeated OS sessions progressively enhance the global efficiency, clustering coefficient, and network density of the neural network while also reducing modularity, leading to a more integrated and cohesive network structure. These structural and functional changes improve the network’s ability to transmit and process information, providing a more robust foundation for reservoir computing tasks. Similarly, Fig. 4 highlights the strengthening of neural connectivity after OS, with the formation of high-intensity connections and the emergence of highly connected hub nodes. These connectivity enhancements further support the network’s capacity for dynamic and high-dimensional representation of input signals, which is critical for efficient FORCE learning and task execution.^52^^–^^54^

The improved initial performance observed during the third OS session in Fig. 7(c) reflects these cumulative network modifications as repeated OS effectively primes the network for learning and task execution.^55^ The increased global efficiency and strengthened connectivity provide a richer reservoir for FORCE learning, allowing for faster alignment between the system’s output and the target signal during the learning phase. Furthermore, the convergence of all OS sessions to similar final proportions in the fixed weights phase underscores the stability and reliability of the reservoir computing system, ensuring robust performance after training.

**Fig. 8:**
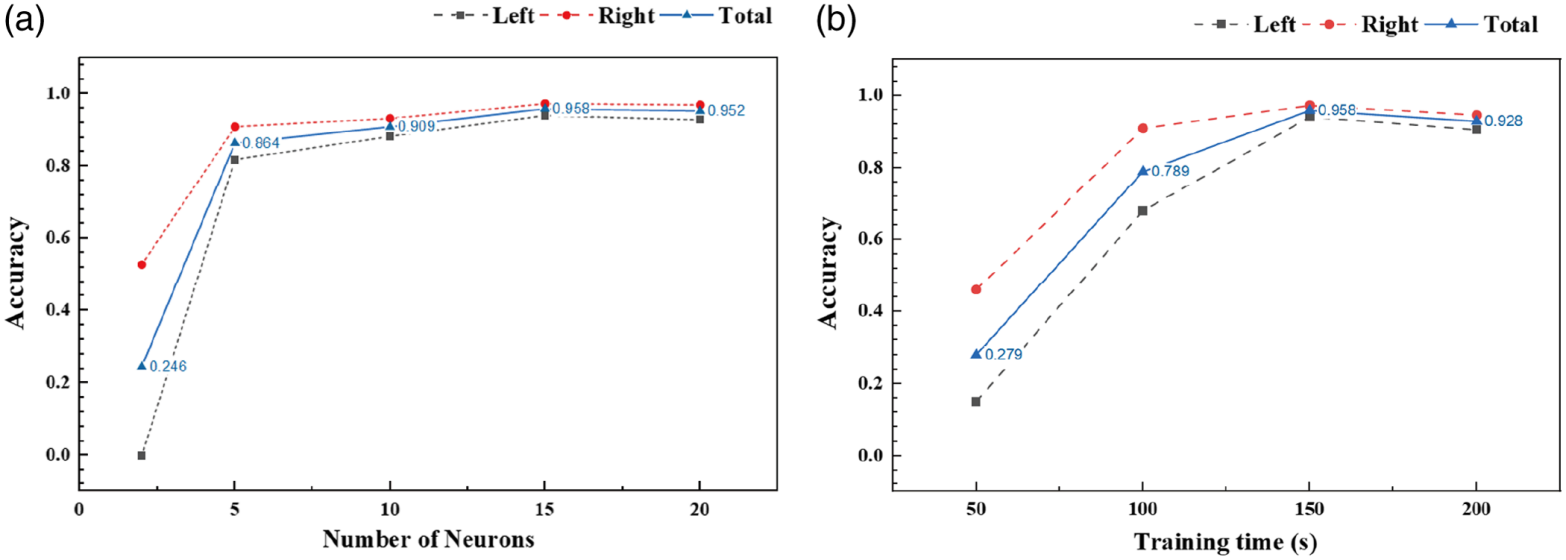
Accuracy of virtual car control based on the number of neurons selected and training time. (a) Accuracy of virtual car control as a function of the number of neurons in the control system, with training time fixed at 150 s. The overall accuracy (blue line) improves with an increasing number of neurons, peaking at 15 neurons with an accuracy of 0.958 before slightly decreasing at 20 neurons. The left (black dashed line) and right (red dashed line) accuracies represent the independent control accuracy of the left and right execution areas, showing similar trends of improvement with increasing neuron numbers. (b) Accuracy of virtual car control as a function of training time, with the number of neurons in the control system fixed at 15. The total accuracy (blue line) increases significantly up to 150 s, reaching an optimal level of 0.958, after which additional training yields minimal improvement. The left (black dashed line) and right (red dashed line) accuracies demonstrate consistent increases during the learning phase, aligning with the overall system performance.

To investigate the relationship between the accuracy of virtual car control and the number of neurons selected as execution units, we conducted a detailed analysis and experiments. As shown in Fig. 8(a), with the training time fixed at 150 s, the accuracy of virtual car control varies with the number of neurons selected as execution units. Here, the accuracy is defined as the ratio of the number of successful obstacle avoidance events by the car to the total number of avoidance trials encountered after training. As the neuron count increases, the overall accuracy improves significantly. With 2 neurons, the total accuracy is only 0.246. The total accuracy rapidly rises to 0.864 with 5 neurons and then slightly increases to 0.909 at 10 neurons. Further increasing the neuron count to 15 and 20 results in total accuracies of 0.958 and 0.952, respectively, indicating that the system achieves optimal performance at ∼15 neurons. These results demonstrate that increasing the number of neurons significantly enhances control accuracy, which stabilizes as the neuron count reaches a certain level.

To further explore the relationship between training time and the accuracy of virtual car control, we performed additional analyses. As depicted in Fig. 8(b), with the number of neurons in the control system fixed at 15, the accuracy of virtual car control also varies with the training time. As the training time extends, accuracy improves substantially, with the total accuracy reaching 0.279 at 50 s and climbing to 0.789 by 100 s. After 150 s, accuracy further stabilizes at 0.958, with minimal gain observed at 200 s (0.928). These findings indicate that the system achieves optimal performance around the 150-s mark, beyond which extended training yields diminishing returns. This suggests that a training duration of ∼150  s is sufficient to maximize control accuracy within the system.

During the experiment, the system encountered 23 obstacles and consistently produced outputs that slightly surpassed the green baseline in nearly every case. This steady performance indicates that the system made precise, timely adjustments throughout. On only one occasion did the output come close to the baseline without exceeding it, reflecting a minor deviation. Despite this, the system achieved a notable obstacle avoidance accuracy of over 95%, highlighting its accuracy and reliability. Its ability to process dynamic inputs with minimal errors demonstrates its effectiveness in complex environments. The near-perfect performance underscores the system’s capacity for rapid adaptation and control. Even during the one deviation, it maintained a trajectory that minimized the impact, showcasing its robustness. These results highlight the system’s capability to perform intricate tasks with precision, making it highly suitable for applications that demand decision-making and effective obstacle navigation.

## 4 Conclusion

The study explored the effects of OS on the biological neural network and evaluated a closed-loop reservoir computing system for obstacle avoidance. Optogenetic stimulation significantly enhanced the network’s structural and functional characteristics, contributing to enhancing the performance of biological PRC tasks. The closed-loop reservoir computing system, using optogenetically stimulated neurons as the reservoir layer, demonstrated effective control of a virtual car in obstacle avoidance tasks. The FORCE learning algorithm facilitated rapid adaptation and stabilization of weights, achieving high alignment between the output and target signals. As the neuron count in the control system and training time increased, the accuracy improved, peaking with 15 neurons and at ∼150  s of training. The system could maintain a high level of accuracy and reliability after training, achieving a remarkable obstacle avoidance success rate of over 95%. This study underscores the potential of optogenetic stimulation in neural modulation and highlights the adaptability of biological reservoir computing systems for complex tasks in neuro-driven control systems.

## 5 Appendix

**Video 1.** Comparison of neuronal network activation under different stimulation patterns. Representative images show the network’s response to optogenetic stimulation using various shapes and optical power densities (OPD). (a) Circular stimulation at $1\;\mathrm{mW}/mm^{2}$. (b) Circular stimulation at $5\;\mathrm{mW}/mm^{2}$. (c) Ring-shaped stimulation at $1\;\mathrm{mW}/mm^{2}$. (MP4, 8.5 MB [URL: https://doi.org/10.1117/1.JBO.30.10.105004.s1]).

**Video 2.** Demonstration of the real-time closed-loop obstacle avoidance task. This video illustrates the three phases of the task: a rapid weight adjustment phase, a slow fine-tuning phase, and a final phase where weight adjustment is paused (fixed weights). After training, the cultured neural network controls the virtual car, achieving an overall obstacle avoidance accuracy of ∼94% (31 out of 33 successful trials). This performance consists of a 100% success rate for left-side obstacles (16/16) and an 88% success rate for right-side obstacles (15/17). (MP4, 10.4 MB [URL: https://doi.org/10.1117/1.JBO.30.10.105004.s2]).

## Supplementary Material

10.1117/1.JBO.30.10.105004.s01

10.1117/1.JBO.30.10.105004.s1

10.1117/1.JBO.30.10.105004.s2

## Data Availability

The data used in this study are freely available upon reasonable request to the authors.
