# Supplementary material for: Optogenetically enhanced physical reservoir computing with in vitro neural networks for obstacle avoidance
Source: J Biomed Opt. 2025 Oct 22;30(10):105004. doi: 10.1117/1.JBO.30.10.105004 (PMC12543164; doi:10.1117/1.JBO.30.10.105004)
Supplement: Supplementary file 1 [file JBO_030_105004_SD001.docx]

Supplementary Material


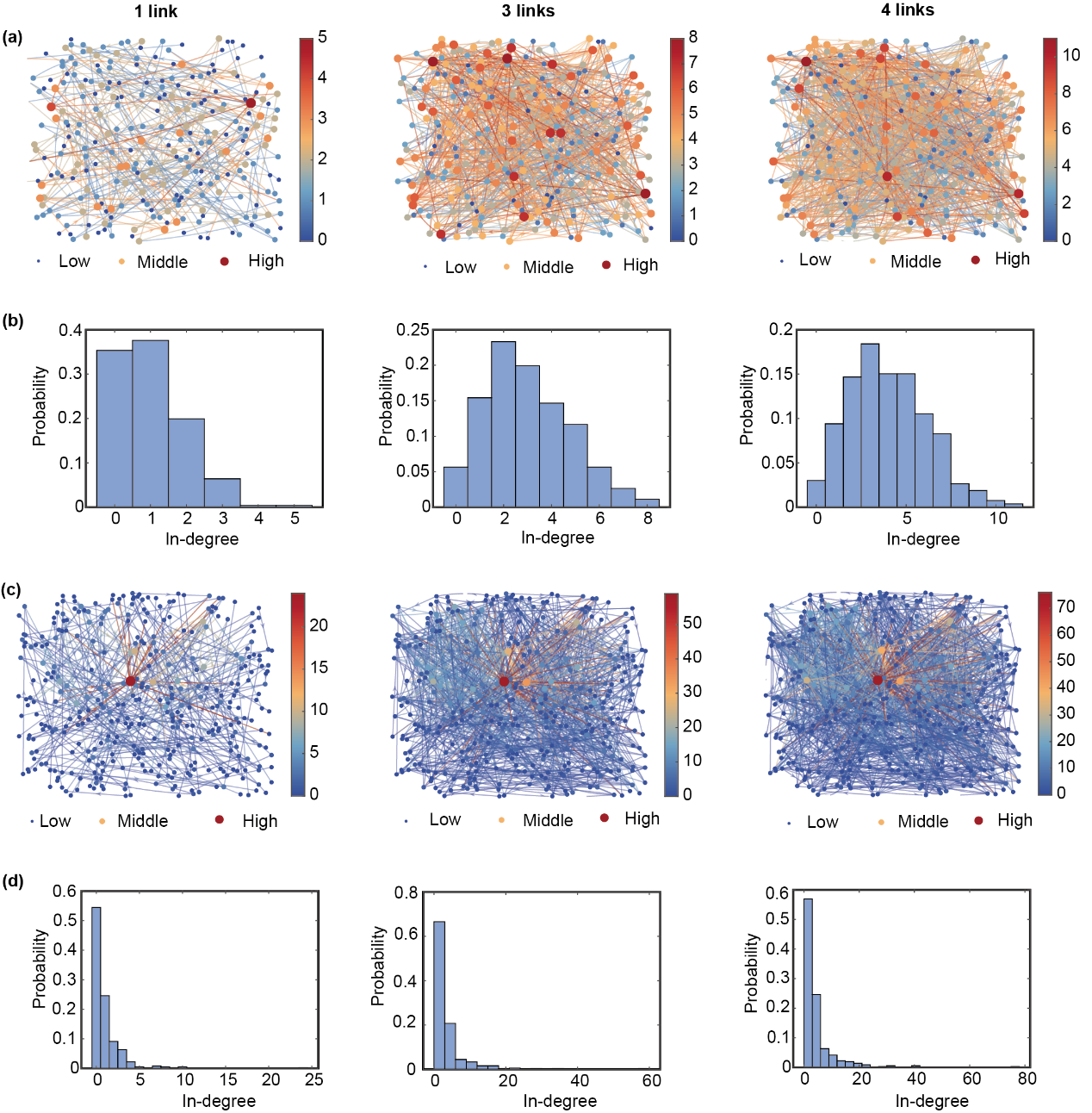


**Fig. S1.** Comparison of network topology and in-degree distributions before and after stimulation for networks with varying connectivity rules. Connectivity maps were constructed by connecting each node to its p most correlated neighbors. Node color corresponds to the in-degree, with warmer colors (e.g., red) indicating a higher number of incoming connections. (a) Network topologies prior to stimulation. From left to right, panels depict networks constructed with p = 1, p = 3, and p = 4, respectively. (b) Corresponding in-degree distributions for the pre-stimulation networks shown in (a). (c) Network topologies following stimulation for p = 1, p = 3, and p = 4 (left to right). (d) Corresponding in-degree distributions for the post-stimulation networks shown in (c).

Figure S2.


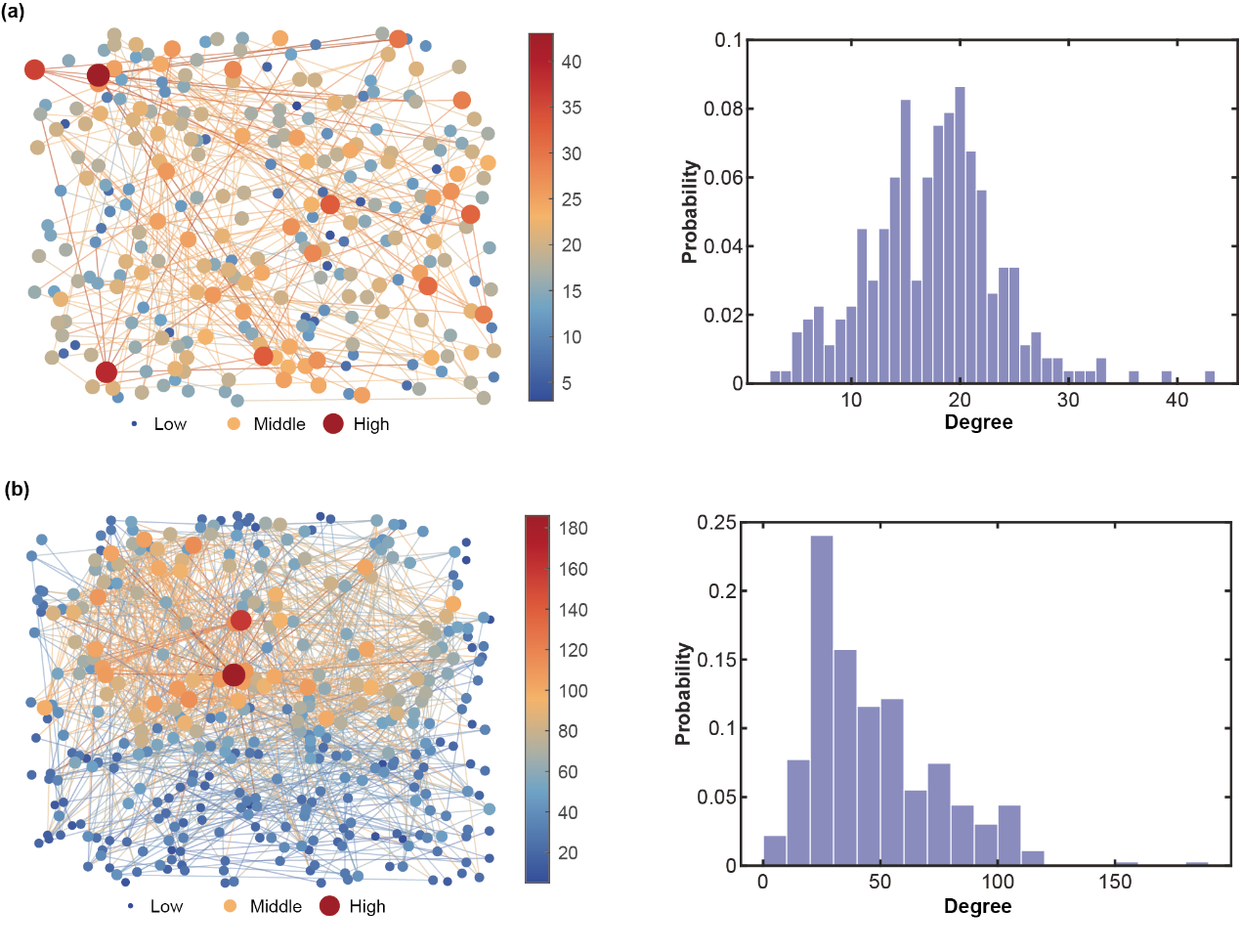


**Fig. S2.** Undirected network topology and degree distribution before and after stimulation. (a) The pre-stimulation state of the network, showing the undirected connectivity map (left) and its corresponding degree distribution (right). (b) The post-stimulation state of the network, showing the undirected connectivity map (left) and its corresponding degree distribution (right). For visual clarity, the connectivity maps in (a) and (b) display a subset representing 10% of the total connections.

*
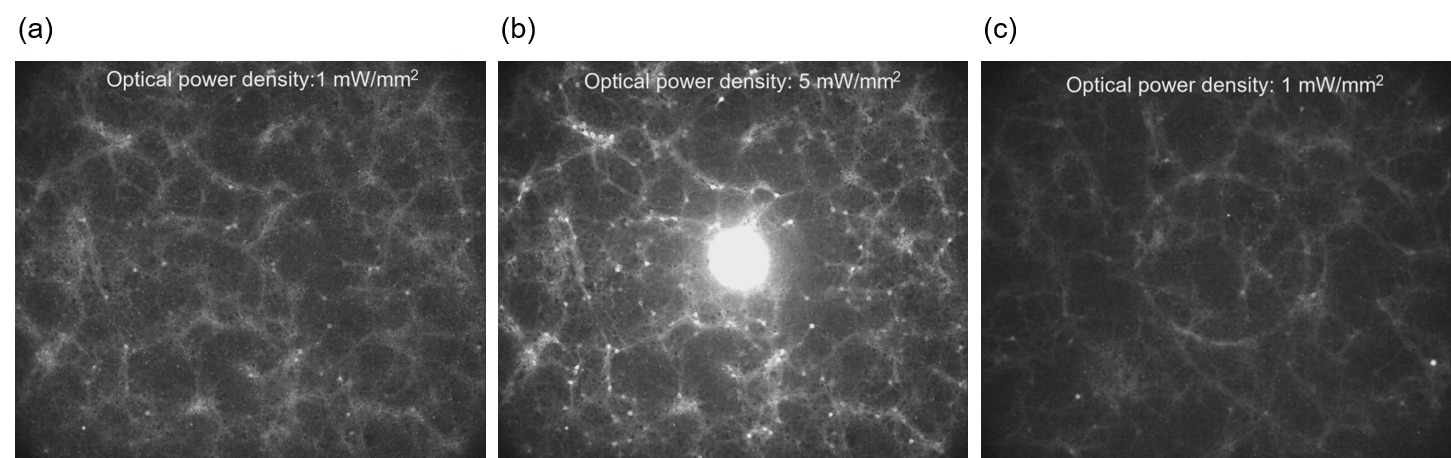
*

**Video 1.** Comparison of neuronal network activation under different stimulation patterns. Representative images show the network's response to optogenetic stimulation using various shapes and optical power densities (OPD). (a) Circular stimulation at 1 mW/mm². (b) Circular stimulation at 5 mW/mm². (c) Ring-shaped stimulation at 1 mW/mm². (MPEG, 8.5 MB)


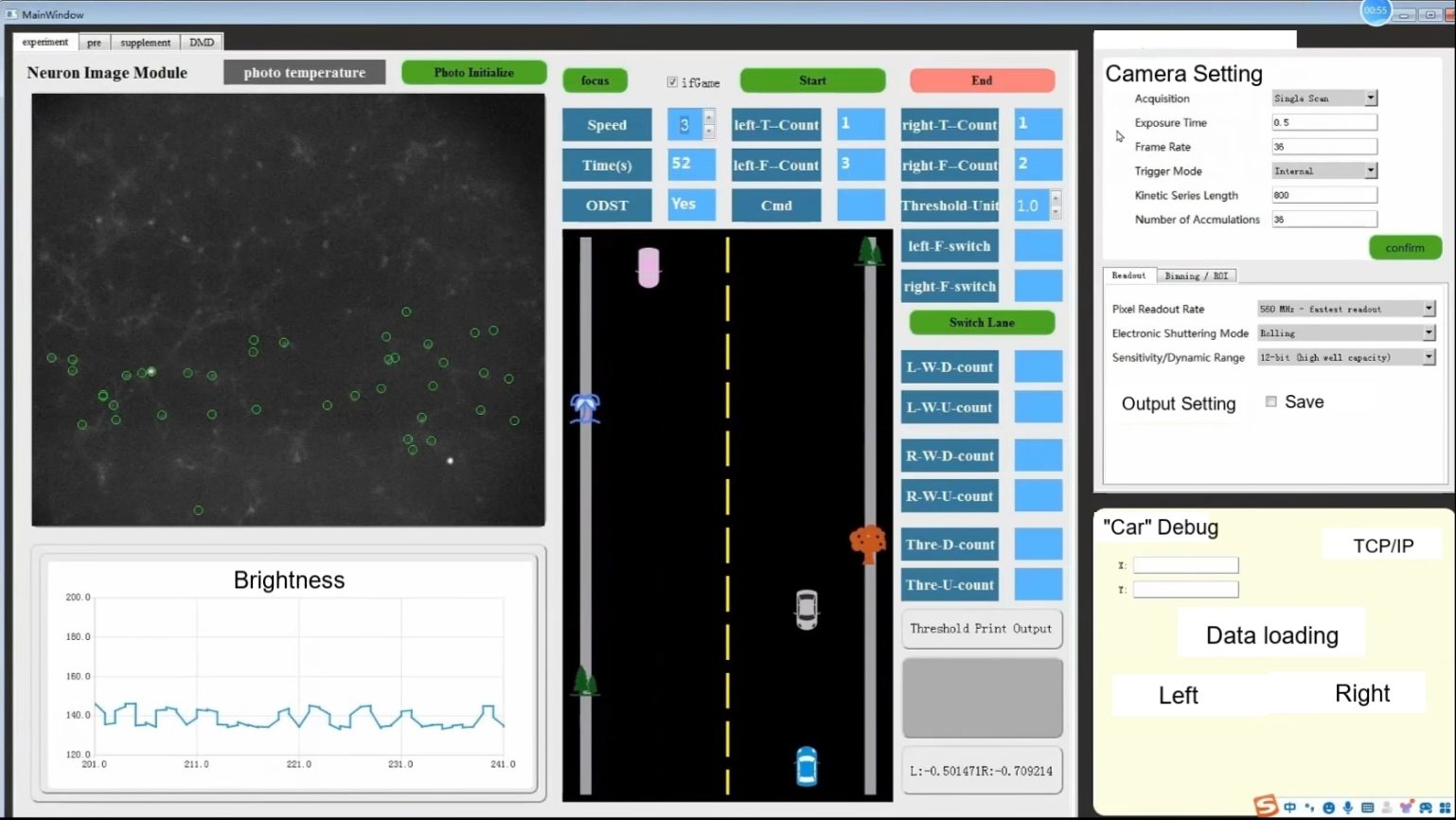


**Video 2.** Demonstration of the real-time closed-loop obstacle avoidance task. This video illustrates the three phases of the task: a rapid weight adjustment phase, a slow fine-tuning phase, and a final phase where weight adjustment is paused (fixed weights). After training, the cultured neural network controls the virtual car, achieving an overall obstacle avoidance accuracy of approximately 94% (31 out of 33 successful trials). This performance consists of a 100% success rate for left-side obstacles (16/16) and an 88% success rate for right-side obstacles (15/17). (MPEG, 10.4 MB)
